# Supplementary material for: Stochastic intracellular replication dynamics shape population-level evolution in Influenza A Virus
Source: Virus Evol. 2026 Aug 5;12(1):veag050. doi: 10.1093/ve/veag050 (PMC13505522; doi:10.1093/ve/veag050)
Supplement: Supplementary file 1 [file supplementary_figures_and_tables_veag050.docx]

# Supplementary information

### **Figure S1. Simulation dynamics.** Temporal dynamics of the **A)** 8 mRNA **, B)** 7 proteins**, C)** 8 vRNPs, and **D)** virions for two different infections with MOI of 1 (blue) and 10 (red). In this example, 2 of the 8 segments are lost to degradation in the infection with MOI of 1, resulting in no virion production and an unsuccessful infection. By contrast, all 8 segments are successfully replicated with an infection of MOI 10 resulting in the production of virions and a successful infection. **E)** Fraction of successful infections as a function of the MOI. In our simulations we consider a successful infection as one in which at least one virion is produced. **F)** Burst size as a function of the MOI. We computed the mean log_10_ burst size to reduce the impact of rare high values on the average. The mean and standard error of the mean are displayed in both plots.

### **Figure S2. Segment specific dynamics.** Dynamics of increase in number of molecules for each genome segment from each infecting virion for an infection with MOI of 10.

### **Figure S3. Lack of correlation between the productivity of the infected cells (total virions produced per cell) and the frequency of the most amplified genome of the initial virions (dominant variant).** Results for MOI 10 are shown. The Pearson correlation coefficient is 0.0185.

### **Table S1.** List of parameters of the model.

| **Parameter** | **Description** | **Value** | **Unit** | **Source or comment** |
| --- | --- | --- | --- | --- |
| kEn | Endocytosis rate. | 0.08 | min^-1^ | Experimentally determined by [(Heldt et al., 2012)](https://www.zotero.org/google-docs/?BpBK8Z) |
| kFus | Rate of fusion to the endosome membrane. | 0.0535 | min^-1^ | Experimentally determined by [(Heldt et al., 2012)](https://www.zotero.org/google-docs/?jZ76Rc) |
| frFus | Fraction of virions that successfully get fused and make it out of the endosome. | 0.51 | dimensionless | Experimentally determined by [(Heldt et al., 2012)](https://www.zotero.org/google-docs/?w0sFCa) |
| kImp | Rate of importation to the nucleus. | 0.1 | min^-1^ | Experimentally determined by [(Babcock et al., 2004)](https://www.zotero.org/google-docs/?Vy7yqU) |
| kM | mRNA transcription rate. | 4166.666667 | nucleotides/min | Experimentally determined by [(Heldt et al., 2012)](https://www.zotero.org/google-docs/?ljV8iD) |
| K_R_ | Polymerase molecules at which transcription rate is half inhibited. | 1.1E7 | molecules | Experimentallly determined by [(Rüdiger et al., 2019)](https://www.zotero.org/google-docs/?hAkStj). |
| kP | Antigenomic synthesis rate. | 0.023 | min^-1^ | Experimentally determined by [(Heldt et al., 2012)](https://www.zotero.org/google-docs/?1gJdeq) |
| kPr | Translation rate. | 1080 | nucleotides/min | [(Spirin, 1986)](https://www.zotero.org/google-docs/?AtHNH8) |
| 1/Drib | Space between two adjacent ribosomes in a single mRNA. | 0.00625 | nucleotides^-1^ | [(Arava et al., 2003)](https://www.zotero.org/google-docs/?Hiiegd) |
| kN | Genomic synthesis rate. | 0.231 | min^-1^ | Experimentally determined by [(Heldt et al., 2012)](https://www.zotero.org/google-docs/?WJZdEP) |
| kBM1 | M1 protein binding rate to RNP. | 2.32E-08 | molecules/min | Experimentally determined by [(Heldt et al., 2012)](https://www.zotero.org/google-docs/?xzBxic) |
| kRel | Virion release rate. | 6.17E-05 | virions/(molecules x min) | Experimentally determined by [(Heldt et al., 2012)](https://www.zotero.org/google-docs/?9CzDOH) |
| 1/L1 | Length of segment 1. | 0.000431034 | nucleotides^-1^ | [(Lamb and Krug, 2001)](https://www.zotero.org/google-docs/?kS18Xh) |
| 1/L2 | Length of segment 2. | 0.000431034 | nucleotides^-1^ | [(Lamb and Krug, 2001)](https://www.zotero.org/google-docs/?K0ATxo) |
| 1/L3 | Length of segment 3. | 0.000452284 | nucleotides^-1^ | [(Lamb and Krug, 2001)](https://www.zotero.org/google-docs/?TH53L9) |
| 1/L4 | Length of segment 4. | 0.000569152 | nucleotides^-1^ | [(Lamb and Krug, 2001)](https://www.zotero.org/google-docs/?9ZWp9G) |
| 1/L5 | Length of segment 5. | 0.000649351 | nucleotides^-1^ | [(Lamb and Krug, 2001)](https://www.zotero.org/google-docs/?m34j0H) |
| 1/L6 | Length of segment 6. | 0.000718391 | nucleotides^-1^ | [(Lamb and Krug, 2001)](https://www.zotero.org/google-docs/?1Vgu4d) |
| 1/L7 | Length of segment 7. | 0.000995025 | nucleotides^-1^ | [(Lamb and Krug, 2001)](https://www.zotero.org/google-docs/?K6HCBp) |
| 1/L8 | Length of segment 8. | 0.001152074 | nucleotides^-1^ | [(Lamb and Krug, 2001)](https://www.zotero.org/google-docs/?8NCXNH) |
| 1-F_Spl7 | Fraction of spliced mRNA 7 for M1 protein. | 0.98 | dimensionless | Inferred by [(Heldt et al., 2012)](https://www.zotero.org/google-docs/?lJzVhg) |
| F_Spl7 | Fraction of spliced mRNA 7 for M2 protein. | 0.02 | dimensionless | Inferred by [(Heldt et al., 2012)](https://www.zotero.org/google-docs/?AMVoCv) |
| F_Spl8 | Fraction of spliced mRNA 8 for NEP protein. | 0.125 | dimensionless | [(Robb et al., 2010)](https://www.zotero.org/google-docs/?37W29m) |
| K_V | Half maximum proteins amount for virion release rate. | 10 | virions | Inferred by [(Heldt et al., 2012)](https://www.zotero.org/google-docs/?sSkhQV) |
| kBRdRp | Polymerase RNA binding rate. | 0.01666667 | molecules/min | Experimentally determined by [(Heldt et al., 2012)](https://www.zotero.org/google-docs/?SNGdlS) |
| kBNP | RNA-pol-complex NP binding rate. | 5.0167E-06 | molecules/min | Experimentally determined by [(Heldt et al., 2012)](https://www.zotero.org/google-docs/?eMnVgy) |
| kExp | RNP-M1 complex exportation rate to the cytoplasm through the binding of NEP. | 1.6667E-08 | molecules/min | Inferred by [(Heldt et al., 2012)](https://www.zotero.org/google-docs/?lWjZE9) |
| RNA_deg | Naked RNA degradation rate. | 0.606 | min^-1^ | Experimentally determined by [(Heldt et al., 2012)](https://www.zotero.org/google-docs/?qQ4B6f) |
| RNAPol_deg | RNA-pol complex degradation rate. | 0.07083333 | min^-1^ | Experimentally determined by [(Heldt et al., 2012)](https://www.zotero.org/google-docs/?xRLlyS) |
| RNPM1c_deg | RNP-M1 complex in the cytoplasm degradation rate. | 0.0015 | min^-1^ | Experimentally determined by [(Heldt et al., 2012)](https://www.zotero.org/google-docs/?sTQecC) |
| RNP_deg | RNP degradation rate. | 0.0015 | min^-1^ | Experimentally determined by [(Heldt et al., 2012)](https://www.zotero.org/google-docs/?n3m9ss) |
| mRNA_deg | mRNA degradation rate. | 0.0055 | min^-1^ | Experimentally determined by [(Heldt et al., 2012)](https://www.zotero.org/google-docs/?QDJCfl) |
| RNPM1n_deg | RNP-M1 complex in the nucleus degradation rate. | 0.0015 | min^-1^ | Experimentally determined by [(Heldt et al., 2012)](https://www.zotero.org/google-docs/?qpPeG0) |
| P_deg | Protein degradation rate. | 0.001357 | min^-1^ | [(Doherty et al., 2009)](https://www.zotero.org/google-docs/?GtJLyU) |
| RdRp | Number of polymerases in a virion. | 37 | molecules | [(Lamb and Krug, 2001)](https://www.zotero.org/google-docs/?qaYjXO) |
| HA | Number of HA proteins in a virion. | 500 | molecules | [(Lamb and Krug, 2001)](https://www.zotero.org/google-docs/?tgIaq1) |
| NP | Number of NP proteins in a virion. | 434 | molecules | [(Lamb and Krug, 2001)](https://www.zotero.org/google-docs/?PLfEqg) |
| NA | Number of NA proteins in a virion. | 100 | molecules | [(Lamb and Krug, 2001)](https://www.zotero.org/google-docs/?jEFzdQ) |
| M1 | Number of M1 proteins in a virion. | 2932 | molecules | [(Lamb and Krug, 2001)](https://www.zotero.org/google-docs/?oLs9aI) |
| M2 | Number of M2 proteins in a virion. | 40 | molecules | [(Lamb and Krug, 2001)](https://www.zotero.org/google-docs/?M4KjiW) |
| NEP | Number of NEP proteins in a virion. | 157 | molecules | [(Lamb and Krug, 2001)](https://www.zotero.org/google-docs/?eQPqgR) |
| IAV mutation rate | IAV mutation rate | 1.35E-5 | mutations per base per generation | [(Aggarwal et al., 2010)](https://www.zotero.org/google-docs/?uITsJC) |
| KA | Viral Polymerase amount at which the cRNA and vRNA production is half maximum | 1E4 | molecules | Used to match the amount of vRNA production of the original experimental results, after introduction the allosteric regulation of vRNA production by free polymerases observed in [(Zhu et al., 2023)](https://www.zotero.org/google-docs/?HSjzQ6) |
| α0 | Fraction of the cRNA and vRNA rate that it is active in the absence of the allosteric activation by viral polymerases | 0.22 | ratio | Observed amount of replication in the absence of free polymerases in [(Zhu et al., 2023)](https://www.zotero.org/google-docs/?YNWBZQ) |
